# Epigenetic Alterations Associated with the Overall Survival and Recurrence Free Survival among Oral Squamous Cell Carcinoma Patients

**DOI:** 10.3390/jcm9041035

**Published:** 2020-04-07

**Authors:** Yasmen Ghantous, Aysar Nashef, Imad Abu-Elnaaj

**Affiliations:** 1 Department of Oral and Maxillofacial Surgery, Baruch Padeh medical center Poriya, The lower Galilee 15208, Israel; dr.aysarn@gmail.com; 2 The Azrieli Faculty of Medicine, Bar Illan University, Safed 1311502, Israel; iabu@poria.health.gov.il

**Keywords:** OSCC survival, OSCC recurrence, methylation biomarkers, TCGA data

## Abstract

Oral squamous cell carcinoma (OSCC) is a fatal disease caused by complex interactions between environmental, genomic, and epigenetic alterations. In the current study, we aimed to identify clusters of genes whose promoter methylation status correlated with various tested clinical features. Molecular datasets of genetic and methylation analysis based on whole-genome sequencing of 159 OSCC patients were obtained from the The Cancer Genome Atlas (TCGA) data portal. Genes were clustered based on their methylation status and were tested for their association with demographic, pathological, and clinical features of the patients. Overall, seven clusters of genes were revealed that showed a significant association with the overall survival/recurrence free survival of patients. The top ranked genes within cluster 4, which showed the worst prognosis, primarily acted as paraneoplastic genes, while the genes within cluster 6 primarily acted as anti-tumor genes. A significant difference was found regarding the mean age in the different clusters. No significant correlation was found between the tumor staging and the different clusters. In conclusion, our result provided a proof-of-principle for the existence of phenotypic diversity among the epigenetic clusters of OSCC and demonstrated the utility of the use epigenetics alterations in devolving new prognostic and therapeutics tools for OSCC patients.

## 1. Introduction

Head and neck cancer rank as the sixth most common malignancy, with a yearly incidence of 830,000 cases worldwide. Compared to other head and neck neoplasms, oral squamous cell carcinoma (OSCC) compromise about 90% of the subtypes within this spectrum with ~40%–50% mortality [1,2]. Research advances in the study of OSCC complexity have revealed that the induction and development of OSCC are due to a sum of genetic changes, epigenetic alterations, and environmental risk factors, especially tobacco, alcohol consumption, and viral infections [3,4,5,6,7].

Due to the heterogeneous nature of oral cancer, the functional and cosmetic results, and the coexistence of frequent medical comorbidities, treatment options of OSCC are evaluated through a multidisciplinary approach, before reaching a final plan for the specific OSCC patient. However, surgical resection with microscopically clear margins of the primary tumor and prophylactic or therapeutic clearance of the lymph nodes, followed by various reconstructive approaches, remains the fundamental treatment for OSCC with adjuvant therapy reserved for high-risk disease [8,9,10,11,12].

Briefly, the term epigenetics describes potentially reversible heritable changes in the genome that are not due to changes in the primary nucleotide sequence of the deoxyribonucleic acid (DNA) itself but rather is due to the interpretation of the genome. The primary mechanisms of epigenetic carcinogenesis involve DNA methylation, histone modifications, and small and non-coding RNAs (ncRNA), which ultimately orchestrate complex gene regulatory pathways [13,14]. In the past few years, it has become apparent that mutations in genes encoding proteins that regulate epigenetic modifications are common in human cancers. Some of these mutations drive tumor initiation, whereas others influence cell growth, immune invasion, metastasis, heterogeneity, and even drug resistance [15]. Taking into account the potential reversibility of these changes, targeting epigenetic alterations is increasingly being recognized as an attractive strategy for cancer therapy [16]. 

Nowadays, several advancements have been made in the development of potentially useful painless and non-invasive diagnostic and prognostic tools for OSCC, based on combined clinical and molecular data. Predicting human cancer-related clusters using a high throughput data sets and genetic and epigenetic network is critical to gain an understanding of disease mechanisms, and is also essential for the development of new diagnostics and therapeutics. Clusters, in general, are of great importance because they not only provide concrete hypotheses about the molecular complexes and signaling pathways, but also offer mechanistic hypotheses about the causes of disease [17]. The usage of clustering algorithms were initially proposed to identify the functional modules or protein complexes in particular phenotypes, and found that disease genes that cause similar diseases exhibit an increased tendency for their protein products to interact with each other. In recent years, many studies have shown the utility of these studies in extracting disease-related clusters/subnetworks and inferring disease-causing genes [17,18,19,20,21,22].

Here, we aimed to identify the clusters of genes whose promoter methylation levels correlated with various tested clinical features, using available clinical and methylation data derived from 157 OSCC patients from The Cancer Genome Atlas (TCGA).

## 2. Methods

Molecular data sets of 528 head and neck carcinoma patients were obtained from the TCGA data portal (https://cancergenome.nih.gov/) [23] and the Genome Data Analysis Center (GDCA). Genomic processing of the molecular data sets was done using cBioPortal for Cancer Genomic analyses (http://www.cbioportal.org/) [24]. 

The molecular datasets included genetic and methylation analysis based on whole-genome sequencing. The human papillomavirus (HPV) status was defined using an empirical definition of >1000 mapped RNA-seq reads, primarily aligning to the viral genes E6 and E7 [25]. The HPV status by mapping of RNA-seq reads was concordant with the genomic, sequencing, and molecular data, and indicated that 36 tumors were HPV-positive and 243 were HPV-negative (To eliminate unnecessary molecular or genetic diversity, only HPV-negative and pathologically-proven oral cavity tumors were included in this study (*n* = 159).

In terms of epigenetic alterations analysis, we classified the samples into consensus clusters, to determine differentially expressed marker genes for each subtype, this way we were able to define the patients into several subgroups, based on genes’ methylation profiles. The clustering analysis for the study cohort was based on data available from the Broad Institute TCGA Genome Data Analysis Center (2016) [24]. The clustering analysis calculated clusters based on a consensus non-negative matrix factorization (NMF) clustering method, which converted the input data set (Table S1) to a non-negative matrix, through column rank normalization and by determining differently expressed major genes into different subtypes. This method was based on an unsupervised learning algorithm that identifies a molecular pattern in complex biological systems, when applied to gene expression data [24]. The top 4160 genes, with maximum standard deviations across beta values, were selected (default cutoff 2). For a better assignment for the sample into the different clusters, the cophenetic correlation coefficients were applied. The reliability for each sample was measured and then assigned to the same cluster across many iterations of the clustering algorithm with random initializations. The consistency for each cluster was determined using the average silhouette values, while the silhouette width was defined as the ratio of the average distance of each sample to the samples in the same cluster to the smallest distance to samples not in the same cluster. If silhouette width was close to 1, it meant that the sample was well-clustered. If silhouette width was close to −1, it meant that the sample was misclassified. The silhouette width was calculated using the R silhouette package [26].

The pathological staging was based on the American Joint Committee on Cancer, 7th edition [27], and overall survival (OS), and recurrence-free survival (RFS) were estimated from the clinically available data using the Kaplan-Meier analysis. Follow-up time was defined as the time that passed from the date of the initial diagnosis, as seen on the pathological report of the biopsy, until either the date of death or the last clinical follow-up, as recorded in the files. The correlation between several clinical parameters (such as pathological staging, alcohol and smoking consumption, gender, race) and promoter genes methylation, to investigate the impact of epigenetic alterations on clinical characteristics. 

### Statistical Analysis

Cross-tab analysis was done to investigate the correlation between clinical parameters and methylation status (cluster-based), using a two-sided Chi-square test. In addition, the association between recurrence and the different clusters was assessed using Fisher’s exact test; *P* value <0.05 was considered to be statistically significant.

## 3. Results

**Figure 1:**
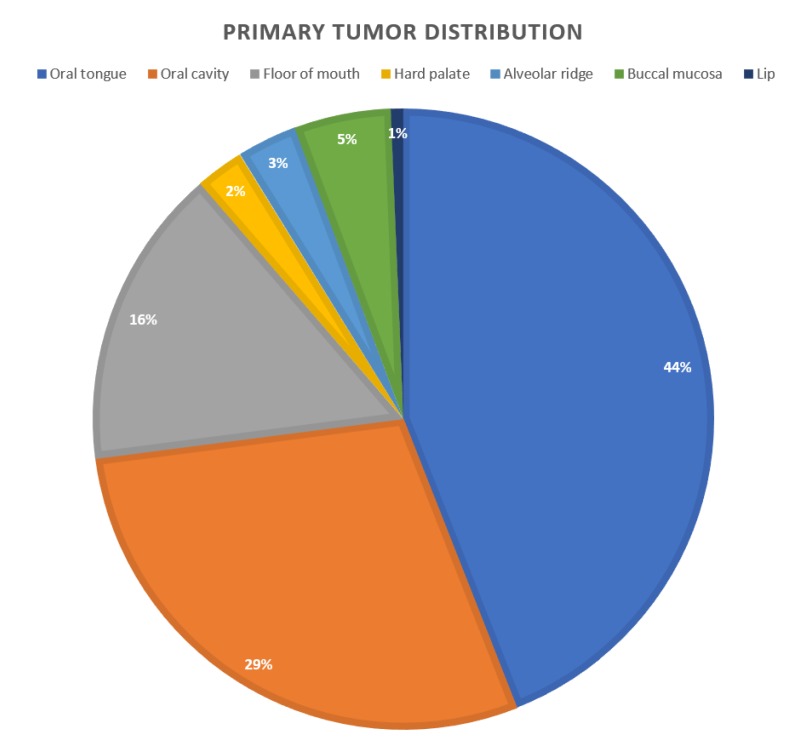
The primary tumor site distribution of the study cohort.

The study cohort included 159 patients, 105 males, and 54 females. The mean age at diagnosis was 62 ± 13 years. Alcohol and tobacco consumption were reported in 63% and 51% of patients, respectively (Table 1). The primary tumor distribution is presented in Figure 1; the tongue was the most common primary tumor site (44%). Based on the aforementioned criteria, 79% of patients had negative margins, 10% had close margins, and 6% had positive margins. Perineural invasion (PNI) was found in 74 (46%) patients of whom only 14 (18%) had local recurrence. Neck dissection (either selective or radical) was performed in 137 (86%) patients. A total of 70 (44%) patients had lymph node metastasis, as seen in the histopathology, with an average of 2 positive lymph nodes for each patient. The mean follow-up period was 26 months. Thirty-eight patients presented with local recurrence (27 male and 11 females), and the average time for recurrence (measured from the day of diagnosis) was 16 months. Clinical parameters that were found to be significant as risk factors for recurrence were—alcohol consumption (*P*-value = 0.01), primary tumors located in the buccal mucosa (*P*-value = 0.03), positive surgical margins (*P*-value = 0.04), and the pathological T staging of the tumor (*P*-value = 0.05). 

### 3.1. Clusters Analysis

**Figure 2:**
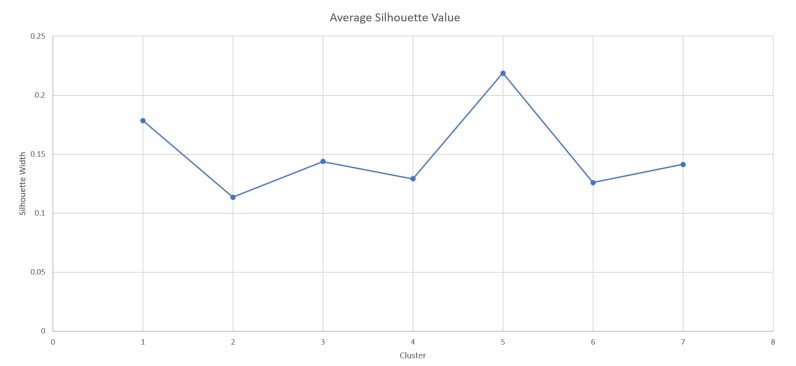
The average silhouette value—the figure shows the average silhouette value ranged between 0.12 and 0.22, for each cluster 1,2,3,4,5,6,7.

Each sample that was included in this study was assigned to a specific cluster based on the promoter methylation of several marker genes. Overall, 4160 major genes were analyzed and were clustered into seven different clusters. Table 2 lists the top 10 methylated genes for each cluster. Each sample was assigned to the most representative cluster, based on core samples that were identified on the basis of the positive silhouette width. Hereby, each cluster included samples with more similarity to the other samples in the same cluster than to any other cluster, using Student’s *t*-test. The consistency for each cluster was determined by the average silhouette values, for each sample, the silhouette width was calculated, and the overall average of all samples was calculated. Figure 2 shows the average silhouette value, ranged between 0.12 and 0.22, for each cluster 1,2,3,4,5,6,7. As shown, the samples showed a well-clustered pattern. 

### 3.2. Correlation between Clusters Pattern and Demographic, Pathological, and Clinical Features

Demographically, the most predominant cluster was cluster 3 (30% of the patients), with a mean age of 67.55 years, the male-to-female ratio in this cluster was 26/20. A significant difference was found regarding mean age in the different clusters, patients belonging to clusters 2 and 6 were significantly younger than patients in the other clusters (*P*-value < 0.05) (Table 3). In terms of pathological parameters (Table 4), no significant correlation was found between the tumor staging and the different cluster. Oral tongue was the predominant primary tumor site in all clusters, except for cluster 4, which showed the worst prognosis in terms of mean survival time and recurrence-free survival (*P*-value = 0.001). 

**Figure 3:**
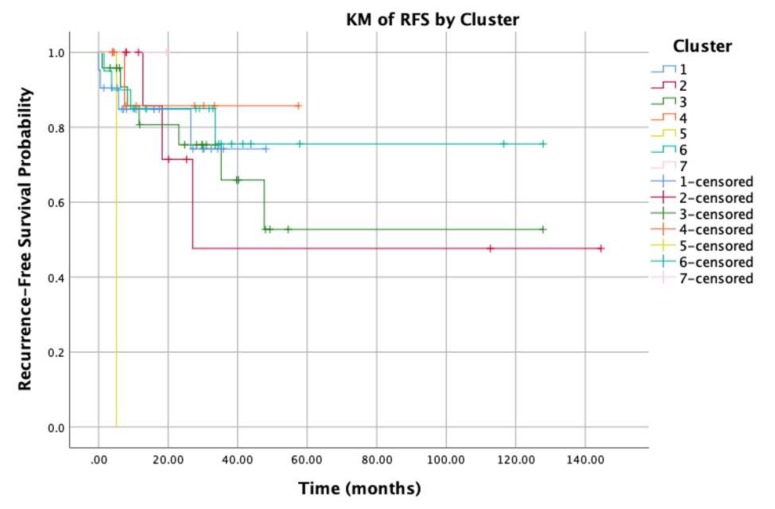
Kaplan-Meier analysis for recurrence free survival of patients.

**Figure 4:**
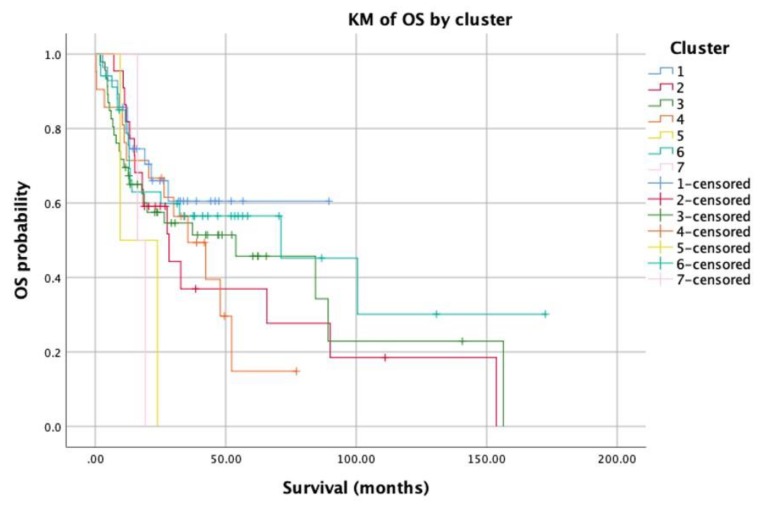
Kaplan-Meier analysis for overall survival of patients.

A pairwise comparison between the different clusters revealed an obvious relation between the different clusters and the overall survival/recurrence free survival of patients (Figure 3 and Figure 4, respectively). The overall mean survival time was 66 months. Nonetheless, the survival time for cluster 6 was significantly higher than the mean survival time of all other clusters, especially clusters 4 and 2 (80 versus 36, 54 months, *P*-value = 0.04). Moreover, cluster 4 showed the worst survival time compared to all other clusters (*P*-value < 0.05). In terms of recurrence-free survival, the overall locoregional recurrence rate was 23%; while cluster 1 showed a recurrence rate of 19%, and in cluster 6 it was 20%. On the other hand, in clusters 2 and 3 the recurrence rate was 28% and 30%, respectively. 

To suggest and pinpoint specific candidate genes, which might be related to OSCC development, we identified the top 5 mythelated genes in both; the worst and the best cluster in terms of mean survival time and recurrence-free survival (i.e., cluster 4 and cluster 6, respectively). The top five mythelated genes in cluster 4 were as follows—*PNMAL*2, *RGS*7*BP*, *GCKR*, *PAX*7, and *IL*2*RA*. The mythelation event resulted in up-regulation and overexpression of all of them, except for the *RGS*7*BP* gene. Interestingly, these genes are well-known for their paraneoplastic mechanism. On the other hand, 3 genes (*ANKRD*11, *BCL*11*A*, and *FOXN*3) out of the top 5 mythelated genes in cluster 6 are well-known in their anti-tumor action (Table 2).

## 4. Discussion

OSCC squamous cell carcinoma is a fatal human disease that undoubtedly remains a health priority, offers significant therapeutic challenges. Although slightly improved prognosis was reported over the past decades, OSCC is still a major public health problem with poor overall 5-year survival rates. Therefore, searching for somatic genetic and epigenetic causes have been of interest among many head and of neck surgeons, oncologists, and oncological researchers. Many laboratories have discovered oncogenes and tumor suppressors for OSCC. However, cumulative evidence revealed more complex mechanisms underlying the development and progression of this disease, including, among others, interactions between genomic and epigenetic alterations [28,29,30,31] Epigenetic alterations are responsible for the regulation of ontologically-related gene expression networks, at an appropriate level of environmental conditions and time, leading to a rise of both normal and diseased phenotype development. 

As the first step toward identifying specific epigenetic alterations at specific genes that might be involved in development and progression of OOSC, we tested the association between demographic, pathological, and clinical features of 159 OSCC patients and different clusters of expressed genes that were significantly different in terms of methylation pattern alterations, using the available high-throughput data of OSCC patients. A significant heterogeneity of the epigenetics landscape in OSCC diseases was observed in the current study, confirmed by the well-clustered pattern in the clustering analysis. 

Interestingly, cluster patterns based on methylation status were to be associated with worse overall survival and recurrence free survival in patients. The survival difference between the two identified clusters, cluster 4 vs. cluster 6, represented a clear difference in the lifetime of these patients. These results fall in agreement with previous reports that showed a significant association between promoter methylation status and worse OSCC patients’ survival [31,32,33,34]. These results demonstrated the utility of epigenetic alterations detection for potential clinical application in OSCC patients. Noteworthy, our data showed that oral tongue was the predominant primary tumor site in all clusters, except for cluster 4, which showed the worst prognosis in terms of mean survival time and recurrence-free survival. Although no correlation was observed between the cancer site and the cluster patterns, this result might indicate that epigenetic alterations are site-related. 

In terms of pathological parameters, our data showed that no significant correlation between the tumor staging or pathological features and the different cluster’s patterns had occurred. This finding is consistent with previous study, which showed a significant correlation between methylation status and worse patient survival, independent of other potential prognostic factors, such as tumor size, lymph node status, clinical stage, and history of tobacco and alcohol use [32]. This finding might indicate that different epigenetic alterations affect independently tumor biology and prognosis, and seemingly indicate that methylation events in particular genes can be used as an independent prognostic factor. We speculate that this might also be explained by the tumorgenesis effect of epigenetic alterations, which alter the normal cell biology and trigger tumorgenesis but have little effect on the tumor microenvironment that directly affects the pathological features of the tumor.

Focusing on the individual gene members of the epigenetic signatures related to the worst and best survival (cluster 4 vs. cluster 6, respectively), we see that genes within cluster 4 are highly relevant to the paraneoplastic genes, while the genes within cluster 6 primarily act as anti-tumor genes. Regarding the suggested genes within the top five regulated gens in cluster 4; gene *PNMAL*2 is a protein coding gene that act as a paraneoplastic Ma antigen [35,36,37]. The next most mythelated gene was in this cluster, *RGS*7*BP*; this gene down-regulation acts as a paraneoplastic alteration. Another gene is *GCKR*, which play a significant role in cancer cell metabolism. The *PAX*7 gene play critical roles during fetal development and cancer growth [38]. Finally, IL2RA is an Interleukin 2 receptor subunit alpha protein involved in suppressing the activity of the immune system against tumor cells [39]. To conclude, the overall epigenetic signature of this cluster is paraneoplastic. On the other hand, 3 out of the 5 top mythelated genes in cluster 6 act as anti-tumor genes. For instance, *ANKRD*11 encodes an ankryin repeat domain-containing protein that inhibits ligand-dependent activation of transcription and prefoliation. *BCL*11*A* gene encodes a C2H2-type zinc-finger protein. During hematopoietic cell differentiation, this gene was down-regulated, and it is believed that this gene was involved in lymphoma pathogenesis, since translocations associated with B-cell malignancies also deregulate its expression. In cluster 6, this gene was down regulated, which might demonstrate its anti-cancer role in this cluster [40]. Finally, *FOXN*3 is a protein-coding gene which has a well-known suppressive role in the progression of colon cancer [41]. To conclude, these results suggest and the nominate number of candidate genes that might be related to the prognosis of the OSCC patients. However, other confirmation and validation studies need to be performed to avoid false positive results.

Indeed, different drugs for human cancers directed at epigenetic modulators have entered clinical development during the last years. These drugs are directed to the three main players of epigenetic modifications; first, the enzymes that place the active and repressive epigenetic marks (writers) (i.e., DNA methyltransferase (DNMT) inhibitors 5-azacytidine (Azacitidine), 5-aza-2′-deoxycytidine (Decitabine), and others; second, those that remove these modifications (erasers) (i.e., histone deacetylase (HDAC) inhibitors); and finally, proteins that recognize the marks (readers). However, except for some agents, the clinical activity of the current epigenetic inhibitors as single agents is largely restricted to hematopoietic malignancies rather than solid cancer type [16,42,43,44,45]. The current results demonstrate the utility of targeting epigenetic modifications for improving the overall survival and recurrence-free survival among OSCC patient. Additionally, it seems that the potential of developing epigenetic agents against our suggested targets should be explored as the basis for rational combinations with other anti-cancer treatment strategies (i.e., chemotherapy, radiotherapy, and immune therapy). Here, we speculate that the epigenome-wide studies associated with OSCC development, in larger cohorts, might help to locate or suggest single targets for OSCC treatment.

As for the limits in the current study; first; as shown, some of the clinical information are missing in some patients, especially when taking into account that 29% of patients missed the information regarding the primary site of disease, which could lead to results of masking and false positive results. Second, the follow up of the patients was still under 5 years and finally; while this study revealed interesting insight into the involvement of epigenetic events during OSCC developments, the confirmation and validation of the data should be further performed in new independent case control cohorts. 

## 5. Conclusions

In conclusion, our result provided a proof-of-principle for the existence of phenotypic diversity among the epigenetic clusters in OSCC, and demonstrated the utility of the use epigenetics alteration in devolving new prognostic and therapeutics tools for OSCC patients. Knowing that staging and pathological systems were imperfect predictors for overall survival and recurrence-free survival among OSCC patients, our results suggest a number of novel genes clusters that could potentially be used as prognostic markers, and in patient selection for adjuvant therapy, following primary surgery treatment. We speculate that, pharmacological manipulation targeting these specific genes might convert the most aggressive tumors into a more benign or manageable counterpart in the clinic, to improve survival.

## Figures and Tables

**Table 1:** Clinical and disease-related data of the study cohort.

Characteristic	Study Cohort	*P*-value (for Recurrence)
Num.	159	
Mean age (± STD)	62 ± 13 years	0.3
Male / Female	105/54	0.4
Tobacco exposure		0.4
Num. (%)	82 (51%)	
Av. Pack/year	47	
Alcohol consumption (%)	101 (63%)	0.01
Primary Tumor Site (%)		0.03
Oral tongue	70 (44%)	
Floor of mouth	25 (15%)	
Buccal Mucosa	7 (4%)	
Alveolar ridge	4 (2%)	
Hard Palate	4 (2%)	
Lip	1 (0.6%)	
Oral Cavity *	25 (15%)	
p N staging (by H&E)		0.4
N0 (%)	81 (50%)	
N1 (%)	35 (22%)	
N2a (%)	2 (1%)	
N2b (%)	20 (12%)	
N2c (%)	12 (7%)	
N3 (%)	1 (0.6%)	
pT staging		0.05
T1 (%)	9 (5%)	
T2 (%)	54 (33%)	
T3 (%)	45 (28%)	
T4a (%)	50 (31%)	
T4b (%)	1 (0.6%)	
TNM staging **		0.7
Stage 1 (%)	7 (4%)	
Stage 2 (%)	40 (25%)	
Stage 3 (%)	41 (26%)	
Stage 4a (%)	69 (43%)	
Stage 4b (%)	2 (1%)	
Surgical Margins status		0.04
Negative margins (%)	126 (79%)	
Positive margins (%)	10 (6%)	
Close margins (%)	16 (10%)	
Overall survival (months)	32 months	0.01

* Unspecified site in oral cavity; ** Pathological staging according to the American Joint Committee on Cancer (AJCC).

**Table 2:** List of top 10 marker genes with *p* ≤ 0.05 (the positive value of the column difference means gene is upregulated in this subtype and vice versa) in each cluster.

Gene	*P*-value	Difference	Cluster
*ATP1A4*	1.57E-51	0.21275	1
*MUC13*	1.45E-37	0.20674	1
*PWRN1*	2.34E-37	0.20661	1
*MIR494*	4.71E-37	0.23066	1
*C3ORF34*	6.24E-36	0.28199	1
*CA5A*	6.78E-36	0.23976	1
*RASA3*	2.53E-35	0.27976	1
*NFATC1*	7.35E-35	0.24022	1
*ATP1A4*	1.57E-51	0.21275	1
*MUC13*	1.45E-37	0.20674	1
*NIT2*	3.24E-62	−0.3497	2
*FAM55C*	4.10E-62	−0.2945	2
*C17ORF75*	1.37E-60	−0.2585	2
*ADAMTS10*	3.23E-58	−0.2637	2
*HES6*	2.66E-56	−0.2276	2
*BLOC1S1*	6.73E-56	−0.2398	2
*GJA4*	3.68E-55	−0.2762	2
*ZC3H12D*	4.33E-55	−0.232	2
*EMX1*	7.11E-54	−0.2292	2
*MYO7A*	1.52E-53	−0.4031	2
*C11ORF49*	2.68E-48	0.24034	3
*HLA−L*	2.73E-48	0.31546	3
*ANKRD11*	3.18E−48	−0.251	3
*RGS17*	2.04E-43	0.26871	3
*LOC441869*	2.34E-43	0.22858	3
*SLC22A3*	2.24E-42	0.22055	3
*HIVEP2*	5.94E-41	−0.2035	3
*CXXC5*	8.96E-41	0.20011	3
*FOXN3*	1.94E-40	0.27933	3
*BDKRB1*	2.53E-40	0.2203	3
*PNMAL2*	4.20E-40	0.3468	4
*RGS7BP*	5.74E-38	−0.1932	4
*GCKR*	8.15E-36	0.25594	4
*PAX7*	1.77E-33	0.34096	4
*IL2RA*	2.05E-32	0.25151	4
*SHC2*	1.03E-29	−0.1744	4
*LTA*	2.97E-29	0.16822	4
*PRRX2*	3.59E-29	0.17614	4
*CCL24*	1.17E-28	0.35067	4
*ODZ2*	2.75E-28	0.28822	4
*ASAP1*	1.37E-50	0.25071	5
*AGPAT4*	6.80E-44	0.2505	5
*ASAP2*	7.60E-40	0.27287	5
*ATG9B*	1.60E-34	−0.2079	5
*ARPC1B*	1.96E-30	−0.2608	5
*APBB2*	2.50E-30	−0.2005	5
*ARSG*	1.54E-29	−0.2827	5
*BHLHE40*	1.01E-22	−0.3006	5
*ANKH*	1.15E-22	0.3368	5
*C3ORF1*	2.62E-22	−0.3024	5
*FOXN3*	6.42E-39	−0.58665	6
*BASP1*	6.65E-37	0.20106	6
*ANKRD11*	4.28E-34	0.214	6
*BIVM*	4.21E-33	−0.2719	6
*BCL11A*	6.62E-31	−0.20825	6
*CCDC88B*	6.10E-30	0.19286	6
*C7ORF20*	8.42E-28	0.22003	6
*ABCA13*	3.15E-27	−0.248	6
*CAPN2*	2.05E-26	−0.2111	6
*LHFPL2*	1.58E-77	−0.2262	7
*PAK6*	4.20E-63	−0.2863	7
*SESN3*	1.13E-52	−0.213	7
*LOC728743*	1.75E-41	−0.1538	7
*GNG3*	8.10E-38	−0.1653	7
*CHRNE*	1.70E-35	−0.2985	7
*PIK3R1*	2.38E-34	−0.1491	7
*LOC150568*	6.66E-31	0.14291	7
*PPIE*	8.88E-31	−0.1251	7
*MEF2B*	5.36E-26	−0.1208	7

**Table 3:** Clusters analysis: The demographic data within each cluster.

Cluster	Num. (%)	Mean Age (years)	M/F Ratio	Smoking (%)	Alcohol
1	29 (18%)	65.44	19/9	12 (43%)	18 (64%)
2	21 (13%)	55.47	17/5	10 (45%)	16 (72%)
3	46 (30%)	67.55	26/20	21 (45%)	27 (59%)
4	21 (13%)	65.95	18/3	7 (33%)	14 (67%)
5	2 (1%)	58	2/0	1 (50%)	2 (100%)
6	34 (21%)	54.52	19/15	22 (65%)	19 (56%)
7	2 (1%)	65.66	2/0	2 (100%)	2 (100%)

**Table 4:** Clusters analysis: The table shows the pathological data characterization of the revealed clusters.

Cluster	1	2	3	4	5	6	7
p T staging:							
T1	4 (14%)	1 (4%)	1 (2%)	2 (9%)	0	0	0
T2	10(35%)	8 (36%)	12 (26%)	7 (33%)	0	16 (47%)	0
T3	5 (18%)	5 (23%)	18 (39%)	5 (24%)	1 (50%)	8 (24%)	1 (50%)
T4a	9 (32%)	8 (36%)	14 (30%)	7 (33%)	1 (50%)	10 (30%)	1 (50%)
T4b	0	0	1 (2%)	0	0	0	0
p N staging:							
N1	9 (40%)	4 (18%)	8 (17%)	3 (14%)	0	10 (29%)	0
N2	8 (27%)	6 (27%)	11 (24%)	5 (24%)	1 (50%)	6 (18%)	2 (100%)
N3	0	0	1 (2%)	0	0	0	0
TNM							
Stage I	4 (14%)	1 (4%)	0	8 (38%)	0	0	0
Stage II	3 (10%)	7 (32%)	11 (24%)	6 (28%)	0	12 (35%)	0
Stage III	6 (21%)	4 (18%)	13 (28%)	5 (24%)	1 (50%)	9 (26%)	0
Stage IVa	15 (54%)	10 (45%)	20 (44%)	0	1 (50%)	13 (38%)	2 (100%)
Stage IVb	0	0	2 (4%)	0	0	0	0
PNI							
Yes	16 (57%)	6 (27%)	21 (45%)	10 (48%)	1 (50%)	19 (56%)	2 (100%)
No	7 (25%)	11 (50%)	18 (39%)	6 (29 %)	1 (50%)	10 (29%)	0
Primary tumor site							
Oral tongue	16 (57%)	11 (50%)	23 (50%)	8 (38%)	1 (50%)	21 (62%)	0
Floor of mouth	3 (10%)	3 (13%)	8 (17%)	5 (24%)	0	4 (12%)	0
Hard palate	3 (10%)	0	1 (2%)	0	0	0	2 (100%)
Alveolar ridge	2 (7%)	0	1 (2%)	0	0	1 (3%)	0
Buccal mucosa	0	2 (9%)	3 (6%)	1 (5%)	1 (50%)	2 (6%)	0
Lip	0	0	0	1 (5%)	0	0	0
Oral cavity	4 (14%)	6 (27%)	10 (21%)	6 (28%)	0	6 (17%)	0

## Supplementary Materials

The following are available online at https://www.mdpi.com/2077-0383/9/4/1035/s1. Table S1: lists of genes per cluster.

## References

[1] Paolino G., Didona D., Macrì G., Calvieri S., Mercuri S.R., Scott J.F., Gerstenblith M.R. (2018). Nasopharyngeal Melanoma. Noncutaneous Melanoma [Internet].

[2] Bray F., Ferlay J., Soerjomataram I., Siegel R.L., Torre L.A., Jemal A. (2018). Global cancer statistics 2018: GLOBOCAN estimates of incidence and mortality worldwide for 36 cancers in 185 countries. CA. Cancer J. Clin..

[3] Lacko M., Braakhuis B.J.M., Sturgis E.M., Boedeker C.C., Suárez C., Rinaldo A., Ferlito A., Takes R.P. (2014). Genetic Susceptibility to Head and Neck Squamous Cell Carcinoma. Int. J. Radiat. Oncol..

[4] Stransky N., Egloff A.M., Tward A.D., Kostic A.D., Cibulskis K., Sivachenko A., Kryukov G.V., Lawrence M., Sougnez C., Mckenna A. (2012). The mutational landscape of head and neck squamous cell carcinoma. Science (80-).

[5] Leemans C.R., Braakhuis B.J.M., Brakenhoff R.H. (2011). The molecular biology of head and neck cancer. Nat. Rev. Cancer.

[6] Lindsay C., Seikaly H., Biron V.L. (2017). Epigenetics of oropharyngeal squamous cell carcinoma: Opportunities for novel chemotherapeutic targets. J. Otolaryngol. Head Neck Surg..

[7] Irimie A.I., Ciocan C., Gulei D., Mehterov N., Atanasov A.G., Dudea D., Berindan-Neagoe I. (2018). Current insights into oral cancer epigenetics. Int. J. Mol. Sci..

[8] Joo Y.H., Cho J.K., Koo B.S., Kwon M., Kwon S.K., Kwon S.Y., Kim M.S., Kim J.K., Kim H., Nam I. (2019). Guidelines for the surgical management of oral cancer: Korean society of thyroid-head and neck surgery. Clin. Exp. Otorhinolaryngol..

[9] Pfister D.G., Spencer S., Brizel D.M., Burtness B., Busse P.M., Caudell J.J., Cmelak A.J., Colevas A.D., Dunphy F., Eisele D.W. (2014). Head and neck cancers, version 2.2014. JNCCN J. Natl. Compr. Cancer Netw..

[10] Di Taranto G., Chen S.H., Elia R., Sitpahul N., Chan J.C.Y., Losco L., Cigna E., Ribuffo D., Chen H.C. (2019). Outcomes following head neck free flap reconstruction requiring interposition vein graft or vascular bridge flap. Head Neck.

[11] Jain P.V., Sharan R., Manikantan K., Clark G.M., Chatterjee S., Mallick I., Roy P., Arun P. (2020). Redefining adequate margins in oral squamous cell carcinoma: Outcomes from close and positive margins. Eur. Arch. Oto-Rhino-Laryngol..

[12] Chi A.C., Day T.A., Neville B.W. (2015). Oral cavity and oropharyngeal squamous cell carcinoma-an update. CA. Cancer J. Clin..

[13] Jenuwein T., Allis C.D. (2001). Translating the histone code. Science (80-).

[14] Berger S.L., Kouzarides T., Shiekhattar R., Shilatifard A. (2009). An operational definition of epigenetics. Genes Dev..

[15] Pfister S.X., Ashworth A. (2017). Marked for death: Targeting epigenetic changes in cancer. Nat. Rev. Drug Discov..

[16] Mohammad H.P., Barbash O., Creasy C.L. (2019). Targeting epigenetic modifications in cancer therapy: Erasing the roadmap to cancer. Nat. Med..

[17] Ideker T., Sharan R. (2008). Protein networks in disease. Genome Res..

[18] Chen J., Aronow B.J., Jegga A.G. (2009). Disease candidate gene identification and prioritization using protein interaction networks. BMC Bioinform..

[19] Franke L., Van Bakel H., Fokkens L., De Jong E.D., Egmont-Petersen M., Wijmenga C. (2006). Reconstruction of a functional human gene network, with an application for prioritizing positional candidate genes. Am. J. Hum. Genet..

[20] Oti M., Snel B., Huynen M.A., Brunner H.G. (2006). Predicting disease genes using protein-protein interactions. J. Med. Genet..

[21] Pujana M.A., Han J.D.J., Starita L.M., Stevens K.N., Tewari M., Ahn J.S., Rennert G., Moreno V., Kirchhoff T., Gold B. (2007). Network modeling links breast cancer susceptibility and centrosome dysfunction. Nat. Genet..

[22] Lim J., Hao T., Shaw C., Patel A.J., Szabó G., Rual J.F., Fisk C.J., Li N., Smolyar A., Hill D.E. (2006). A Protein-Protein Interaction Network for Human Inherited Ataxias and Disorders of Purkinje Cell Degeneration. Cell.

[23] The Cancer Genome Atlas data portal. https://www.cancer.gov.

[24] Broad Genepattern: NMFConsensus. https://cloud.genepattern.org.

[25] Lawrence M.S., Sougnez C., Lichtenstein L., Cibulskis K., Lander E., Gabriel S.B., Getz G., Ally A., Balasundaram M., Birol I. (2015). Comprehensive genomic characterization of head and neck squamous cell carcinomas. Nature.

[26] R Silhouette Package. http://stat.ethz.ch/R-manual/R-patched/library/cluster/html/silhouette.html.

[27] Edge S.B., Compton C.C. (2010). The american joint committee on cancer: The 7th edition of the AJCC cancer staging manual and the future of TNM. Ann. Surg. Oncol..

[28] Naganuma K., Hatta M., Ikebe T., Yamazaki J. (2014). Epigenetic alterations of the keratin 13 gene in oral squamous cell carcinoma. BMC Cancer.

[29] Chen C., Zhang Y., Loomis M.M., Upton M.P., Lohavanichbutr P., Houck J.R., Doody D.R., Mendez E., Futran N., Schwartz S.M. (2015). Genome-wide loss of heterozygosity and DNA copy number aberration in HPVNegative oral squamous cell carcinoma and their associations with disease-specific survival. PLoS ONE.

[30] Melchers L.J., Clausen M.J.A.M., Mastik M.F., Slagter-Menkema L., Van Der Wal J.E., Wisman G.B.A., Roodenburg J.L.N., Schuuring E. (2015). Identification of methylation markers for the prediction of nodal metastasis in oral and oropharyngeal squamous cell carcinoma. Epigenetics.

[31] Ribeiro I.P., Caramelo F., Esteves L., Oliveira C., Marques F., Barroso L., Melo J.B., Carreira I.M. (2018). Genomic and epigenetic signatures associated with survival rate in oral squamous cell carcinoma patients. J. Cancer.

[32] Ai L., Vo Q.N., Zuo C., Li L., Ling W., Suen J.Y., Hanna E., Brown K.D., Fan C.Y. (2004). Ataxia-Telangiectasia-Mutated (ATM) Gene in Head and Neck Squamous Cell Carcinoma: Promoter Hypermethylation with Clinical Correlation in 100 Cases. Cancer Epidemiol. Biomarkers Prev..

[33] Ribeiro I.P., Caramelo F., Marques F., Domingues A., Mesquita M., Barroso L., Prazeres H., Julião M.J., Baptista I.P., Ferreira A. (2016). WT1, MSH6, GATA5 and PAX5 as epigenetic oral squamous cell carcinoma biomarkers—A short report. Cell. Oncol..

[34] Hayashi M., Guerrero-Preston R., Sidransky D., Kochz W.M. (2015). Paired box 5 methylation detection by droplet digital PCR for ultra-sensitive deep surgical margins analysis of head and neck squamous cell carcinoma. Cancer Prev. Res..

[35] Lipp J.J., Marvin M.C., Shokat K.M., Guthrie C. (2015). SR protein kinases promote splicing of nonconsensus introns. Nat. Struct. Mol. Biol..

[36] Kimura K., Wakamatsu A., Suzuki Y., Ota T., Nishikawa T., Yamashita R., Yamamoto J., Sekine M., Tsuritani K., Wakaguri H. (2006). Diversification of transcriptional modulation: Large-scale identification and characterization of putative alternative promoters of human genes. Genome Res..

[37] Dunham A., Matthews L.H., Burton J., Ashurst J.L., Howe K.L., Ashcroft K.J., Beare D.M., Burford D.C., Hunt S.E., Griffiths-Jones S. (2004). The DNA sequence and analysis of human chromosome 13. Nature.

[38] Toki S., Wakai S., Sekimizu M., Mori T., Ichikawa H., Kawai A., Yoshida A. (2018). PAX7 immunohistochemical evaluation of Ewing sarcoma and other small round cell tumours. Histopathology.

[39] Jia Z., Zhang Z., Yang Q., Deng C., Li D., Ren L. (2019). Effect of IL2RA and IL2RB gene polymorphisms on lung cancer risk. Int. Immunopharmacol..

[40] Satterwhite E., Sonoki T., Willis T.G., Harder L., Nowak R., Arriola E.L., Liu H., Price H.P., Gesk S., Steinemann D. (2001). The BCL11 gene family: Involvement of BCL11A in lymphoid malignancies. Blood.

[41] Dai Y., Wang M., Wu H., Xiao M., Liu H., Zhang D. (2017). Loss of FOXN3 in colon cancer activates beta-catenin/TCF signaling and promotes the growth and migration of cancer cells. Oncotarget.

[42] Lübbert M., Suciu S., Hagemeijer A., Rüter B., Platzbecker U., Giagounidis A., Selleslag D., Labar B., Germing U., Salih H.R. (2016). Decitabine improves progression-free survival in older high-risk MDS patients with multiple autosomal monosomies: Results of a subgroup analysis of the randomized phase III study 06011 of the EORTC Leukemia Cooperative Group and German MDS Study Group. Ann. Hematol..

[43] Fenaux P., Mufti G.J., Hellström-Lindberg E., Santini V., Gattermann N., Germing U., Sanz G., List A.F., Gore S., Seymour J.F. (2010). Azacitidine prolongs overall survival compared with conventional care regimens in elderly patients with low bone marrow blast count acute myeloid leukemia. J. Clin. Oncol..

[44] Stewart D.J., Issa J.P., Kurzrock R., Nunez M.I., Jelinek J., Hong D., Oki Y., Guo Z., Gupta S., Wistuba I.I. (2009). Decitabine effect on tumor global DNA methylation and other parameters in a phase I trial in refractory solid tumors and lymphomas. Clin. Cancer Res..

[45] Fenaux P., Mufti G.J., Hellstrom-Lindberg E., Santini V., Finelli C., Giagounidis A., Schoch R., Gattermann N., Sanz G., List A. (2009). Efficacy of azacitidine compared with that of conventional care regimens in the treatment of higher-risk myelodysplastic syndromes: A randomised, open-label, phase III study. Lancet Oncol..

